# HPV Vaccination: Predicting Its Effect on Cervical Cancer Rates

**DOI:** 10.1371/journal.pmed.0030202

**Published:** 2006-04-04

Each year, nearly 500,000 new cases of cervical cancer are diagnosed around the world, and more than 250,000 women die from the disease. Most of these cases occur in developing countries where there is no routine screening for precancerous lesions. By contrast, in developed countries, national screening programs have greatly reduced the number of women dying from this cancer—between 1955 and 1992 in the US, for example, cervical cancer deaths dropped by 74%.

Infection with a sexually transmitted human papillomavirus (HPV) is a precondition for the development of cervical cancer. Of the 35 HPV types that can infect the genital tract, about half have oncogenic potential—the rest cause benign warts. The immune system clears most HPV infections but persistent infection with HPV type 16 accounts for approximately 55% of cervical cancers. Because of the strong association between cervical cancer and HPV infection, several HPV type-specific vaccines are being developed. Early results suggest that these vaccines can prevent almost 100% of persistent infections with the relevant HPV type, raising the possibility of reducing the incidence of cervical cancer by prophylactic vaccination. But what would the impact of such vaccines be in countries that already have cervical cancer screening programs? To find out, Ruanne Barnabas and colleagues have developed a dynamic transmission model of HPV 16 infection and progression to cervical cancer using epidemiological data from Finland. Their analyses indicate that high coverage of women alone over many decades with a vaccine that provides long-term protection would greatly reduce type-specific cancer incidence, a reduction that would be maximized by combining vaccination with routine screening.

The researchers' model is represented by a flow chart in which susceptible women acquire an HPV infection that, in most cases, is cleared by their immune system. In some women, persistent infection induces precancerous lesions that can progress to invasive cervical cancer, regress spontaneously, or be screened and treated. HPV infection in men is represented by a simpler flow chart—they simply become infected and then develop immunity. The researchers incorporated values for parameters such as sexual activity, screening protocols, and treatment rates obtained from published Finnish studies in their model and calibrated it using historical data on the proportion of the Finnish population with antibodies to HPV 16.

To allow them to model how vaccination will affect the incidence of cervical cancer, Barnabas and colleagues first estimated the transmission probability of HPV in the Finnish population. This probability provides a measure of how easily HPV spreads—if it were 1.0, every sexual partnership a woman had with a man infected with HPV would result in her also becoming infected. The researchers' transmission probability estimate of 0.6 is high, which indicates that universal coverage with a very effective vaccine will be needed to eliminate HPV infection in the population. The researchers put this value (which is subject to great uncertainty) and estimated values for age at sexual debut and the annual number of sexual partners into their model; they also assumed that vaccination takes place before sexual debut, is 100% effective, and gives life-long protection.

The model predicts that vaccinating both men and women will be little better than vaccinating women alone, irrespective of whether vaccine coverage is high or low. Furthermore, although vaccinating 90% of young women before sexual debut could decrease HPV type-specific cervical cancer incidence by 91%, delaying vaccination until after sexual debut could decrease the impact of vaccination. The model also predicts that if 90% of women were vaccinated without screening, there would be 0.6 cases of cervical cancer per 100,000 women per year (compared with seven out of 100,000 with no intervention); vaccination plus screening every five years would reduce this incidence further, by two-thirds. Finally, the researchers investigated how the duration of vaccine-conferred protection might affect invasive cervical cancer rates. The model predicts that—unintuitively—short-lived protection will marginally increase cervical cancer rates compared with no vaccination if, as some people believe, older women are more susceptible to the persistent HPV infections that progress to cervical cancer than are younger women. Booster vaccinations would avoid this potential problem.

The researchers conclude that the most effective strategy for the reduction of cervical cancer in developed countries in which incidence is already low is widespread vaccine coverage (both in terms of the HPV type targeted and the fraction of the population vaccinated), combined with current screening protocols. Whether this recommendation is adopted will depend on how vaccines perform in ongoing phase III trials and on a detailed economic assessment of the options available.

## 

**Figure:**
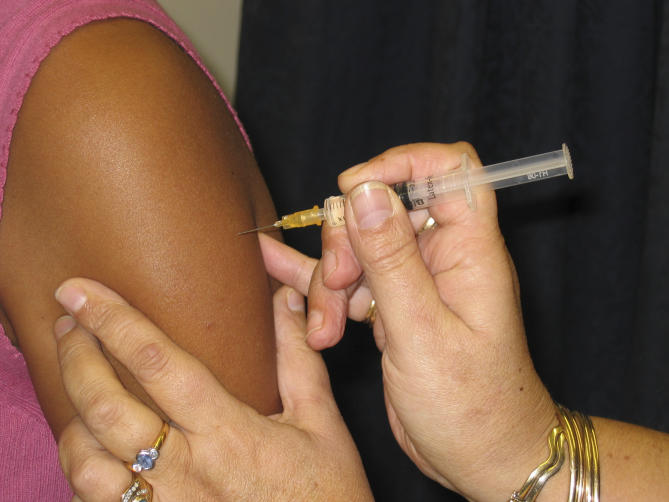
There is a high percentage of HOXB4GFP ^+^ cells early after transplantation

